# Urinary iodine: comparison of a simple method for its determination in microplates with measurement by inductively-coupled plasma mass spectrometry

**DOI:** 10.1038/srep39835

**Published:** 2017-01-03

**Authors:** Michael Haap, Heinz Jürgen Roth, Thomas Huber, Helmut Dittmann, Richard Wahl

**Affiliations:** 1 Internal Medicine IV (Endocrinology, Diabetology, Angiology, Nephrology, and Clinical Chemistry), Eberhard-Karls-University, Tübingen, Germany; 2 Labor Dr. Limbach & Kollegen, GbR, Medizinisches Versorgungszentrum, Im Breitspiel 15, 69126 Heidelberg, Germany; 3 Department of Nuclear Medicine, Eberhard-Karls-University, Tübingen, Germany

## Abstract

The aim of our study was to develop and validate an inexpensive, rapid, easy to use quantitative method to determine urinary iodine without major procurement costs for equipment. The rationale behind introducing this method is the increasing demand for urinary iodine assessments. Our study included 103 patients (76 female, 27 male), age (arithmetic mean) 52 ± 17.3 years. Urinary iodine was determined in microplates by a modification of the Sandell-Kolthoff reaction. The results were compared with inductively-coupled plasma mass spectrometry (ICP-MS) for iodine, considered as reference method. Geometric mean of urinary iodine determined by the Sandell-Kolthoff reaction method was 62.69 μg/l (95% confidence interval 53.16–73.92) whereas by the ICP-MS method it was 65.53 μg/l (95% confidence interval 54.77–78.41). Passing-Bablok regression equations for both methods gave y = 3.374 + 0.873x (y: Sandell-Kolthoff method, x: ICP-MS). Spearman´s correlation coefficient was 0.981, indicating a very high degree of agreement between the two methods. Bland-Altman plots showed no significant systematic difference between the two methods. The modified Sandell-Kolthoff method using microtiter plate technique presented here is a simple, inexpensive semi-automated method to determine urinary iodine with very little toxic waste. Comparison with the ICP-MS-technique yielded a good agreement between the two methods.

Iodine is an essential micronutrient and the primary element of thyroid hormones and can normally be obtained by consumption of foods that contain it or to which it is added, e.g. as iodized salt^1^. The recommended daily intake is 150 μg for nonpregnant adults, 220 μg for pregnant women, and 90 to 120 μg for children aged 1 to 13 years^2^. Metabolic iodine balance studies have shown that fecal excretion of iodine is usually negligible^3^,^4^. It is well established that in a state of iodine sufficiency approximately 90% of the ingested iodine is excreted in the urine^5^,^6^. In iodine deficiency lower fractions can be excreted. Therefore, urinary iodine (UI) can be used as a tool to evaluate the status of iodine nutrition of a population^7^ and serves as a sensitive parameter of recent iodine intake which reflects the equilibrium between intake and excretion^8^. UI acts as a marker of iodine intake over a few days. This indicator is often used in epidemiologic studies to assess the iodine supply^9^,^10^. A comprehensive review is given in the WHO-UNICEF-ICCIDD guide for program managers^11^. UI is usually expressed in μg/dl or μg/l, rather than in SI units (μmol/l), and World Health Organization guidelines use μg/l^12^. In spot urine samples iodine is preferentially quantified in relation to urinary creatinine excretion^13^. UI measured as 24 h excretion in μg/d is a highly reliable measurement of individual iodine excretion and thereby iodine intake^13^. Results can also be expressed in relation to urinary creatinine excretion. Different techniques for the determination of urinary iodine, such as chemical methods, paired-ion reversed-phase HPLC or inductively-coupled plasma mass spectrometry (ICP-MS) have been developed over the years [for an overview, see Jooste *et al*.^14^]. Most of the analytical methods for determination of UI concentrations are based on spectrophotometric measurements of the Sandell-Kolthoff-reaction^15^,^16^. This procedure, which measures total iodine, relies on the iodine catalyzed reduction reaction of yellow tetra-ammonium cerium (IV) sulfate to the colorless cerous form by arsenite. It can be executed manually or can be automated to different degrees, for example, using microplate methods as described by Ohashi *et al*.^17^, Grimm *et al*.^18^, Hedayati *et al*.^19^ or Tang *et al*.^20^ on the basis of the Sandell-Kolthoff-reaction. An automated method using the development of a flow-injection analysis technique for on-line catalytic digestion and spectrophotometric detection of UI by Sandell-Kolthoff-reaction was described by Yaping *et al*.^21^, Gnat *et al*.^22^ describe a fast colorimetric method for UI determination using a modified Sandell-Kolthoff-reaction with ferroine/arsenious acid. Their method is semiquantitative. All tubes, including calibrators and samples are ranked in order of color change. However, iodine determination on a protein-bound iodine (PBI-) AutoAnalyzer® (Technicon Instruments, Chauncey New York) is no longer available. Instead, the technically challenging ICP-MS method is employed^23^,^24^, considered the most accurate for measuring urinary iodine, and often taken as the reference method by the Centers for Disease Control and Prevention (CDC)^25^. WHO currently still recommends the Sandell-Kolthoff-method for epidemiological studies^26^.

Selecting between the different methods to determine UI depends on the intended application, the number of samples, technical capabilities and the financial possibilities of the laboratory. Here, we describe a simple, inexpensive, semi-automated urinary iodine determination method based on the Sandell-Kolthoff-reaction which can be performed in microtiter plates. We compare the results to those determined by an ICP-MS-method.

There is an increasing demand for urinary iodine determination. Worldwide the most common cause of goiter is iodine deficiency^27^. Iodine deficiency is linked to many adverse effects on growth and development and to mental retardation consequent to fetal hypothyroidism^27^,^28^. Our method described here may therefore be applied as a first step in monitoring those studies recently proposed by a consortium of European researchers intended to optimize programs for preventing iodine deficiency disorders^29^ and could be applied to monitor population iodine status worldwide. On the other hand iodine excess may provoke iodine hyperthyroidism or exacerbate hypothyroidism in patients with underlying autoimmune thyroiditis^30^ or may increase the risk of autoimmune thyroid disease^31^. Iodine excess has a negative influence on radioiodine therapy and on radionuclide thyroid imaging^30^,^32^. Thus, UI determination is also an important methodology in the individual-based assessment of thyroid disorders.

## Results

### Patient characteristics

The study included 103 patients (76 female and 27 male) ranging in age from 15 to 83 years (arithmetic mean ± standard deviation (sd) = 52 ± 17.3 years) who were referred to the Department of Nuclear Medicine, University of Tübingen, Germany, to evaluate their iodine status. In the following ‘internal’ refers to our applied Sandell-Kolthoff-method and ‘ICP-MS’ to the mass spectrometric determination.

### Recovery testing

Recovery testing (n = 10) of iodine added to the standard calibrators at 203, at 102 and at 51 μg/l was between 96% and 115%. When a sample has an unusually high iodine concentration (e.g. iodinated contrast media or povidone iodine) this can be seen mostly by the substantially weaker decolorization reaction in the microtiter plate at the end of incubation and reflected by recovery values outside the accepted range. The corresponding sample must not only be diluted, but the determination of the whole microtiter plate has to be repeated, since this contamination does not rule out cross-contamination in adjacent wells. If it is known in advance that samples have an unusual high iodine content, e.g. by contrast media, then a dilution (1:5 or 1:10) in advance is strongly recommended.

### Precision

#### Repeatability (intra-assay precision)

Low iodine content, standard calibrator 13 μg/l: coefficient of variation (CV) 19.2% (12.3 ± 2.4 μg/l; n = 10). Low iodine content, standard calibrator 25 μg/l: CV 14.2% (24.2 ± 3.4 μg/l; n = 10). Medium iodine content, standard calibrator 103 μg/l: CV 5.5% (102.1 ± 5.6 μg/l; n = 10). High iodine content, standard calibrator 203 μg/l: CV 5.1% (206.7 ± 10.6 μg/l; n = 10). In parentheses, mean ± sd and number of samples are given.

#### Intermediate Precision

Within-laboratory variation with a urine bench quality control; aliquots stored at −18 °C): CV 10.7% (109.2 ± 11.7 μg/l; n = 10). In parenthesis mean ± sd and number of samples are given.

### Interfering substances

There are numerous studies on substances such as L-ascorbic acid or potassium thiocyanate which can interfere with the Sandell-Kolthoff- method^17^,^33^. On the other hand, it is also known that a preceding strong acidic ashing can prevent such interference in the Sandell-Kolthoff-reaction^17^,^34^. Because we have applied a strong acid ashing, we have dispensed with the investigation of such interfering substances.

### Analysis of the measurements

**Figure 1:**
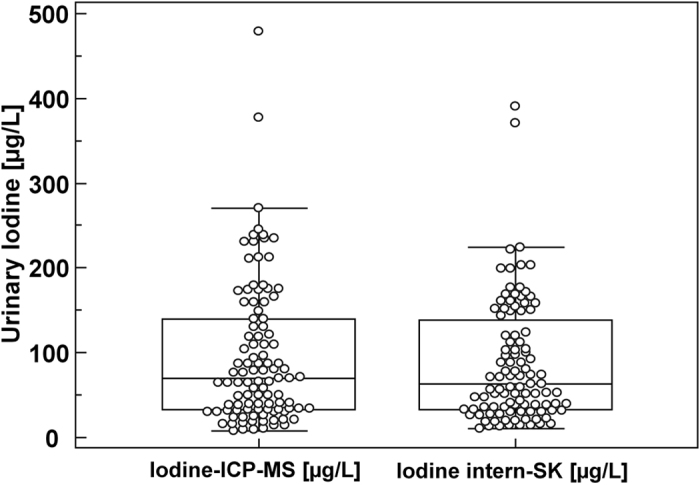
Box-and-whisker plots of the urinary iodine concentrations measured by the ICP-MS method and by the internal method. Sample size n = 103 each. Each box in the figure represents the interquartile range (25^th^ and 75^th^ percentiles); the line inside each box is the median value. The whiskers represent the range of data up to a limit of 1.5-fold above or below the interquartile range; individual points are displayed for data exceeding the 1.5-fold limit. The two outside values (outliers) are displayed as separate circles. Box-and-whisker key numbers: 
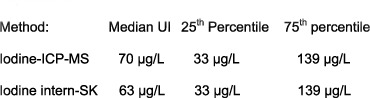

Data obtained with either method are normally distributed after logarithmic transformation. Fig. 1 shows the distribution of urinary iodine in box-and-whisker plot format. The UI of our internal method ranged from 11–391 μg/l (geometric mean: 62.96; 95% confidence interval (CI) of the mean 53.16–73.92). Corresponding values from the ICP-MS method showed similar results with UI concentrations ranging from 8–479 μg/l (geometric mean: 65.53; 95% CI of the mean 54.77–78.41).

**Figure 2:**
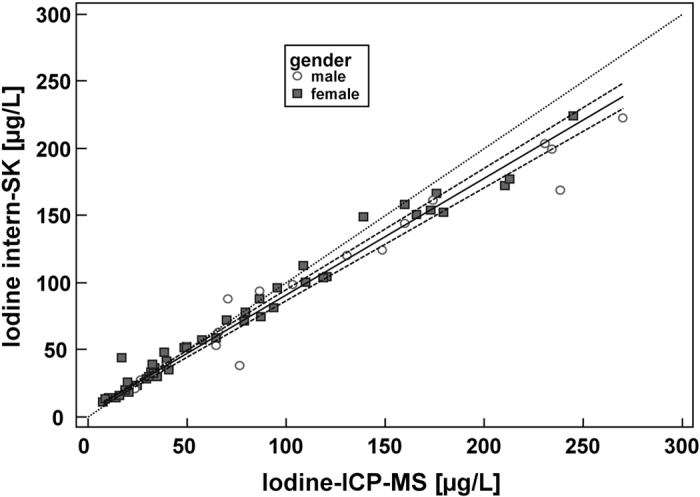
Comparison of the ICP-MS method versus the Sandell-Kolthoff method by Passing-Bablok regression. Two sampels (>300 μg/L) out of n = 103 each are way out of the others in terms of their UI and are not shown in this diagram. Excluding these very high values did not alter the results significantly. The scatter diagram shows the regression line (solid line), the 95% confidence interval for the regression line (dashed lines) and identity line (x = y, dotted line). The regression equation for n = 101 each is y = 3.374 + 0.873x. (The regression equation for n = 103 each, not shown here, is y = 3.373 + 0.873x). Variable y: ‘internal iodine’, variable x: ‘ICP-MS iodine’. Constant differences were evaluated by calculating the intercept of the regression within the 95% CI (intercept: 3.373, CI: 1.964–4.722). The slope is 0.873, the 95% CI 0.844–0.905. Spearman’s coefficient of rank correlation = 0.981. Significance level P < 0.0001. In summary, no bias between the two methods and no significant deviation from linearity could be detected.

Passing-Bablok regression (Fig. 2) showed a very high correlation between the two methods (no bias and no significant deviation in linearity). The Passing-Bablok regression equation for both methods was y = 3.374 + 0.873x (y: Sandell-Kolthoff-method, x: ICP-MS). Spearman´s correlation coefficient was 0.981.

**Figure 3:**
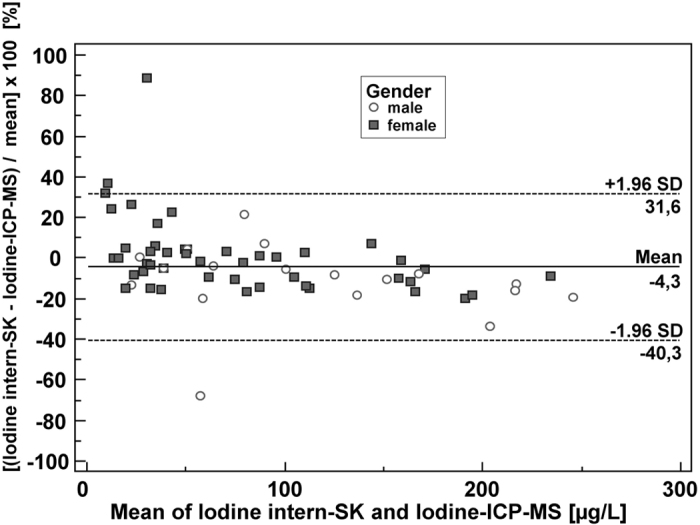
Bland-Altman plot to compare our two measurement techniques for iodine determination. Sample size shown is n = 101 each. Two samples (outliers) out of n = 103 with very high UI values (>300 μg/l) are excluded in this plot, but this revision of the number of patients did not affect the overall results as compared to the plot with n = 103 (not shown). Differences between the Sandell-Kolthoff method [μg/l] and ICP-MS [μg/l], expressed as percentages of the geometric means of the paired values on the y-axis *vs*. the geometric mean [μg/l] of the two measurements on the x-axis are plotted. (y-axis: [geometric mean Sandell-Kolthoff method (μg/l) – geometric mean ICP-MS method (μg/l)/geometric mean (μg/l)] × 100 [%]).Horizontal lines show the mean difference and the 95% CI of limits of agreement (confidence limits of the bias), which are defined as the mean difference plus/minus 1.96 times the standard deviation of the differences. The mean differences are near the 0-line. Bias: −4.3%. In summary, no significant systematic difference between the two methods can be discerned.

The Bland-Altmann plot (Fig. 3) for urinary iodine concentrations determined by the ICP-MS-method and the ‘internal’ method shows that the mean difference line is almost at the 0-line.

## Discussion

We have improved and simplified the existing Sandell-Kolthoff-reaction method for urine iodine determination and adapted it to our existing laboratory conditions. Our modified method is time-saving, cost-effective and environmentally conscious. The aim was to measure the physiological concentrations of iodine in urine reliably. The range of our standard curve covers iodine deficiency and sufficient iodine intake according to WHO epidemiological criteria for assessing iodine nutrition based on median UI concentrations in school-age children^6^,^35^,^36^. Due to a potential risk of cross-contamination in adjacent wells the standard curve of our Sandell-Kolthoff-method cannot be arbitrarily extended. For population-based studies in the 10 countries with excessive iodine intake and a median urinary iodine concentration >300 μg/l^6^, the samples should be diluted as a precautionary measure. In 1937, Sandell and Kolthoff described a sensitive procedure for the determination of quantities of iodide of the order of 0.05 to 3 γ (μg) in 1 ml solution or in a suitable amount of solid sample (sodium chloride), known in the literature as the Sandell-Kolthoff-reaction or method^37^. This now traditional method is widely used as a simple, sensitive and reliable technique for iodine determination in biological materials including urine. However, for reliable determination in biological specimens, digestion of the sample is required as a first step. Over the years, modifications of this method have been developed. In 1993, Dunn *et al*. reported that the pre-digestion of urine samples with chloric acid resulted in transformation of all iodo-compounds into iodine and prevented the formation of substances which interfere with the subsequent redox reaction^34^. In 1996, a less hazardous method was published by Pino *et al*., who replaced chloric acid with persulfate digestion^38^. Again, this approach was also not ideal in that it produced a great deal of toxic waste and was very time-consuming. The first microplate-based method exploiting the Sandell-Kolthoff-reaction was developed by Ohashi *et al*. in 2000^17^. For the digestion procedure these authors used a microtiter plate placed into a special stainless steel cassette. For heating in an oven at 110 °C the cassettes had to be sealed tightly so that no acid vapours could escape. This step, together with the risk of condensation of vapour on the top of the wells of the microplate during cooling the cassettes with tap water to room temperature could be a technical flaw. By using a multichannel-pipette the authors could greatly increase daily sample throughput. In addition, the amount of toxic waste was much reduced.

The microplate method of Tang *et al*.^20^ used no special sealing cassettes for the digestion process, but instead digested urine samples in special microplates at 95 °C for only 30 minutes in a PCR thermal cycler. These microplates were sealed by an adhesive film and by compression pads. In order to document the quality of their method, the authors compared it with the ICP-MS method and found good agreement between the results of the two techniques when using external quality assurance samples. However, in our opinion, it cannot be concluded from this comparison that the digestion of urine samples by their method was in fact optimal, because quality assurance samples may behave differently from actual urine. Urine contains substances which can interfere with the Sandell Kolthoff-reaction if they are not oxidized by adequate digestion. This special problem does not exist in the ICP-MS-method. Grimm *et al*. used a similar microplate procedure but digested the urine specimens for 45 minutes in a PCR thermal cycler^18^. They also found a good correlation with the ICP-MS reference method. Tight sealing of the microplates for heating in a PCR thermal cycler and then their cooling to room temperature by avoiding water condensation might be a critical step in this digestion procedure. Hedayati *et al*.^19^ also use a microplate method for UI determination but describe a different digestion method using a household microwave oven at 440 W power for 10 minutes as the optimal digestion condition. The results of these authors were not compared with an ICP-MS- method.

Our method represents an adaptation based on the use of microtiter plates which can be easily applied in any laboratory equipped with an acid-resistant fume hood (which could be substituted by a fume hood with trap as described by Dunn *et al*.^34^ when no special fume cupboard is available). Our procedure is simpler and does not require specially designed stainless steel cassettes and Teflon-laminated silicon rubber gaskets to seal microplates. This special device may not be easy to procure in some countries^23^. We digested the specimens in heat-resistant tubes (reusable after rinsing) in the fume hood and could therefore almost double the incineration temperature. This allows a better digestion of the specimens in only 25 min instead of 60 min as is the case with the method of Ohasi *et al*.^17^. However, there is some risk in daily routine that the microplate digestion method of these authors does not work reliably because condensation of vapour on the tops of wells and cross contamination cannot be completely excluded despite a Teflon rubber gasket. Moreover, our method requires only one microplate per batch for measuring absorbance, whereas the method of Ohasi *et al*.^17^ and the method of Tang *et al*.^20^ requires separate microtiter plates: polypropylene microtiter plates for ashing and polystyrene microtiter plates for measuring absorbance. The procedure described here can be carried out in a reasonable time. No purchasing of expensive special equipment is required and the chemicals used are inexpensive. Therefore, a widespread use of this method is realistic. The performance characteristics of our method show that our procedure gives sufficiently accurate and precise results compared to the Inductively Coupled Plasma Mass Spectrometry approach used as the reference method. The comparison is surprising. In the study by Marcours *et al*.^39^ ICP-MS yielded slightly, but significantly lower urinary iodine values than the Sandell-Kolthoff-method. Because a comparison with the ICP-MS method was not available when introducing our method, the recovery testing was especially important for us. We found that the recovery did not exceed the predetermined limit of acceptance of ≤15%. A population’s iodine status is defined as ‘inadequate’ if the median UI concentration is <100 μg/l according to the World Health Organization and the median urinary iodine concentrations <20 μg/l indicate severe iodine deficiency in a population^26^. For our lowest standard (13 μg/l) the CV exceeds the set of limit of ≤15%. From the medical point of view this imprecision is not critical because the diagnosis of severe iodine deficiency is unaffected.

The limiting factor of the throughput capacity of our method is the number of boreholes in the metal block thermostat. However, such heating blocks are commercially available in different sizes that can be matched to the daily sample throughput requirements.

The good agreement between our method and the reference method on the same sample material shows that there is no detrimental interference in the results. This is of fundamental importance in the handling of urine samples and in the evaluation of the method. Especially methods using milder digestion can cause interference with substances in the urine that lead to incorrect results^33^,^40^. The application of acid digestion steps for removing interfering substances was originally designed for measurement of iodine in blood or serum^33^,^41^. Therefore, these applied digestion steps should thoroughly remove common interfering substances in the more dilute urine matrix 12.

Due to the historical nature of our comparison study, quality controls were not -as would have been optimally desirable- compared to each other for this iodine determination method. However, this should not affect the overall results.

In conclusion, the study described here documents the successful establishment of a modified method for the determination of urinary iodine based on the use of microtiter plates. Our measurement method and the PC-assisted evaluation of the 96-well plates by a microplate reader represents a significant gain in time, provides the advantage of calculating quality parameters and provides online results. Moreover, the transfer of the Sandell-Kolthoff-method to microtiter plates reduces the consumption of chemicals, lowers costs and produces significantly less toxic waste compared to the classical method. In addition our method could be applied to monitor population and individual iodine status worldwide. ICP-MS reference measurements have confirmed the high quality of our method.

## Materials and Methods

### Equipment

Bench-mounted fume cupboard for handling acids (exchangeable by a fume hood with trap as described by Dunn *et al*.^34^ when no special fume cupboard is available).

Vortex mixer (Vortex Genie, Scientific Industries, Inc., Bohemia, NY 11716, USA).

#### Magnetic stirrer with magnetic stir bar.

 Adjustable pipettes (Eppendorf, Hamburg, Germany): Multipette M4 and Combitips (Eppendorf, Hamburg, Germany) (for quick reaction volume addition).

Thick-walled (1.0–1.2 mm) thermal shock resistant test tubes (outside-d x h = 16 × 130 mm) with beaded rim (26 130 16 07 Duran, Duran Group GmbH, Mainz, Germany).

Metal block thermostat (temperature range 200 °C, accuracy borehole depth 65 mm, diameter 16.2 mm), Liebisch, Bielefeld, Germany.

Polystyrene microplates with flat bottom, plate geometry 8 × 12 wells (655 101 Greiner bio-one, Greiner, Nürtingen, Germany).

CULTURA® M Mini-Incubator (Merck, Darmstadt, Germany).

Microplate spectrophotometer (PowerWaveXS, BioTek®, BioTek Instruments, Inc., Winooski, VT, USA).

### Chemicals

All chemicals were of analytical grade

Deionized HPLC-water (115333.2500 LiChrosov®. Merck,

Darmstadt, Germany) was used for preparation of reagents

Sulfuric acid (95–97%) (9316.2, Roth, Karsruhe, Germany).

Perchloric acid 70–72% (100519.1000, Merck, Darmstadt, Germany)

Nitric acid (65%) (100452.1000, Merck, Darmstadt, Germany)

Sodium chloride (purest) (106404.0500, Merck, Darmstadt, Germany)

Sodium hydroxide tablets (106495.0250, Merck, Darmstadt, Germany)

Arsenic (III) oxide (A 1010, Sigma-Aldrich, Taufkirchen, Germany

Tetra-ammonium cerium (IV) sulfate dihydrate (102273, Merck, Darmstadt, Germany)

Potassium iodate (105051.0100, Merck, Darmstadt, Germany)

Glucose solution 50%) (Glucosteril® 50%, Fresenius-Kabi, Bad Homburg, Germany

Urine bench quality control (prepared by collecting urine, stored in aliquots at −18° C in polypropylene tubes).

### Solutions

#### Acid solution for ashing

The working acid should always be freshly prepared with 4 parts of perchloric acid and 1 part nitric acid (the latter being necessary to give the mixture such a high oxidation potential that no iodide or iodine can escape). The amount of freshly prepared working acid required depends on the size of the batch.

#### Arsenious acid solution (0.125 M)

##### Preparation based on the method of Yaping *et al*. 21

24.8 g arsenic trioxide is dissolved in 500 ml NaOH (0.3 N) while vigorously stirring and gently warming (40 °C) on a magnetic stirrer for approximately 2 days in a closed flask in a fume cupboard. If necessary, low turbidity level can be filtered off (Whatman paper I) before adding sulfuric acid. When the solution is clear, 10 ml sulfuric acid (96%) is carefully added. After the solution has cooled, 30.0 g of sodium chloride is added and the mixture adjusted to a final volume of 1000 ml (chloride in the arsenious acid solution prevents the oxidation of iodide to iodate. Iodate has only a small catalytic effect within the Sandell-Kolthoff-reaction). The prepared solution is stored in a brown glass bottle and is stable for months.

#### Ceric ammonium solution

First, 14 g of tetra-ammonium cerium (IV) sulfate dihydrate are dissolved in 300 ml deionized water in a 1000-ml-volumetric flask. 52 ml sulfuric acid (96%) are then added, with cooling and shaking until the solution is homogeneous. Finally the volume is made up to 1000 ml with deionized water. The flask is stored at room temperature and wrapped in aluminium foil to protect against light. The solution is stable for months.

#### Iodine calibrators

Potassium iodate (I-content = 59.30%) was preferred over KI because it is more stable. In a 1000 ml-volumetric flask, 3.423 g potassium iodate is dissolved in deionized water to prepare a 16 000 nmol/l solution (stock iodine standard). This stock solution is stored in a brown bottle at 7 °C in the refrigerator. The stock solution is stable for months. Serial dilutions with deionized water to make further working standard calibrators (1600; 800; 400; 200; 100; 50; 25 nmol/l corresponding to 203; 102; 51; 25; 13; 6; 3 μg/l iodine) are made freshly for each determination. Because the iodine concentrations are logarithmically graded for the standard curve, there is no zero standard calibrator.

### Procedure

Consecutive series of urine samples (5 ml) from patients (n = 103) referred to the Department of Nuclear Medicine were collected, put into urine cups and kept frozen at −18 °C until processing by a modification of the traditional Sandell-Kolthoff-method in the Division of Clinical Chemistry, Fourth Department of Internal Medicine, University of Tübingen. Iodine-contaminated samples were excluded as far as possible, taking into account the patients’ medical history. Previously screened urine samples by urine dipsticks were also excluded because of the not well known risk of iodine contamination by the sticks. Their glucose area is impregnated with the enzyms glucose oxidase and peroxidase together with colourless potassium iodide as the chromogen which can be oxidized to brown iodine. After immersion of the dipsticks iodide is eluted 42. However in order to minimize the number of unknown iodine contaminations by urine specimens with an iodine content outside the standard curve the urine samples were diluted with water (in general 1:1) prior to measurement.

Seeking a rapid, reliable and accurate method for the determination of urinary iodine, the Department of Nuclear Medicine sent independent aliquots of the same urine samples to the Division of Clinical Chemistry and to a laboratory in Heidelberg, where urinary iodine is assessed routinely using of the highly sophisticated inductively- coupled mass spectrometry (ICP-MS) method, which is currently regarded as the reference method. Examination of the patients’ samples was performed as part of routine diagnostics. Neither of the involved laboratories was aware of the tests being carried out by the other. Finally the observation of the Department of Nuclear Medicine that the results from both laboratories are in agreement for each individual specimen became the incentive for the present study. To blind the samples for the comparison of methods reported here, individual-related data were anonymized not matched as in the two previous statements.

According to German legislation and internal Standard Operating Procedures (SOPs) the quality management of routine diagnostic procedures does not require approval of the local Institutional Review Board (IRB).

### Experimental procedure for our applied Sandell-Kolthoff-method

The present method for iodine determination is based on the colorimetric Sandell-Kolthoff-reaction 21. This depends on the reduction of ceric (IV) sulfate by arsenite in the presence of iodide. Prior to the colorimetric analysis, step (a) is carried out in a bench-mounted fume cupboard for handling acids to vent any fumes liberated. Standard calibrators are treated in the same way as patients’ urine samples.

- Digestion of urine samples to remove interfering substances by oxidation: Interfering substances in urine may contribute positively to the iodine catalytic reaction in the Sandell-Kolthoff-method^37^,^38^. Urine samples of patients, urine bench quality control and not matrix-matched iodate standard calibrators (200 μl each) are pipetted into thick-walled thermal shock-resistant test tubes with beaded rims positioned in a test tube rack. Then 20 μl of glucose solution and 1 ml sulfuric acid per tube are added to prevent protein precipitation. After vortexing, 250 μl ‘acid solution for ashing’ per tube is added as a strong oxidizing agent. After vortexing again, the tubes are put into a metal block thermostat (borehole depth 65 mm) for 25 minutes at 200 °C (boiling point of HNO_3_ 83 °C, of HClO_4_ 203 °C and of H_2_SO_4_ 337 °C). Nitric acid vaporizes with the water. At the same time, breakdown of the perchloric acid oxidizes the sample material. These are then cooled for 5 minutes as a slush. Thereafter, 1 ml/tube of deionized water is added and the solutions mixed. Because our laboratory has good experience in handling perchloric acid digestion for measuring iodine content in foods, we have retained this proven method also for UI determination instead of ammonium persulfate. Pino *et al*.^41^ have advocated ammonium persulfate as a safe alternative oxidizing reagent for measuring urinary iodine, but this digestion is not necessarily applicable to other sample types.
- Catalytic procedure: This is accomplished in 96-well clear polystyrene flat-bottomed microplates for precise optical measurement (working volume 340 μl). solution of ashed patient samples or standards (100 μl each) are pipetted into each well, and 100 μl of arsenious acid solution/well then added.

After rapid addition of 50 μl of yellow ceric (IV) ammonium sulfate solution to all wells and shaking the microtiter plate to start a continuous decolorization reaction, the microplates, sealed with a cap mat, are allowed to stand for a fixed time (20 minutes) at 37 °C in a mini-incubator the translucent plastic door of which must be darkened by aluminium foil because the reaction is light-sensitive.

Absorbance of the remaining Ce (IV), which represents the amount of iodine present in the sample, is then measured with a single channel absorbance microplate spectrophotometer (PowerWaveXS, BioTek®) at a primary wavelength of 405 nm and a reference wavelength of 620 nm. Iodine concentrations are determined automatically by the instrument’s software. For this, the average absorbance value for each set of reference standard and each set of samples is calculated. A standard curve is constructed by plotting the mean absorbance (Y-axis, linear) obtained for each reference standard against its concentration nmol/l (μg/l) (x-axis, log); interpolation of data points by a ‘cubic spline’ curve.

The number of samples that can be determined at the same time is limited by the number of boreholes in the fixed metal block thermostat.

Each of the 8 standard calibrators were analyzed in duplicate per assay. Each patient sample was prepared in 8 replicates. Four of these were used for quadruplicate iodine determination per patient and four for iodine recovery testing per patient to estimate proportional systematic error that can be caused by the presence of interfering substances in the urine. Recovery also served for quality control because no commercial quality controls were available. To this end, the respective specimens were each supplemented with 20 μl of the highest standard calibrator corresponding to 1/10 volume of urine sample. Maximum permissible variation of recovery was set ± 15%. Since we have been working routinely with the method of iodine recovery testing, we used no urine bench quality control material in general.

A supplementary online figure shows the arrangement of standard and patient samples in the microtiter plate. The second set of standards can also be placed in the last row of the plate instead of the second row. This has the advantage that it corrects for variations caused by any delayed pipetting.

## ICP-MS reference method

### Equipment

Quadrupole Inductively Coupled Plasma Mass Spectrometry (X-Series 2 ICP-MS – Thermo-Fisher Scientific, Bremen, Germany).

### Chemicals

Ultra-pure water (Milli-Q® Integral water purification system; Millipore-Merck, Darmstadt, Germany).

EDTA (Ethylenediaminetetraacetic acid, 431788-100 G, Sigma-Aldrich, Taufkirchen, Germany.

Ammonia solution 25%, suprapure (105428.0250, Merck, Darmstadt, Germany.

IC-Anion standard iodide 1.000 g iodide/l (basic compound KI in water) traceable to NIST (National Institute of Standards and Technology), 04301.0000, Bernd Kraft GmbH, Duisburg, Germany. This standard was stored at 4 °C; dilutions of this stock were used for instrument calibration and were prepared freshly for each run. The calibration was carried out linearly by 5 standard solutions (50 μg/l; 100 μg/l; 250 μg/l; 500 μg/l and 1000 μg/l) prepared in ultrapure water.

ICP standard Rhodium, 1.000 g Rh/l (basic compound RhCl3 in HCl 1 mol/l, traceable to NIST (National Institute of Standards and Technology), 03736.0000, Bernd Kraft GmbH, Duisburg, Germany. Stock solutions (stability 1 week at 2–8 °C) and working solutions (freshly produced for each run) were prepared in ultrapure water. Rhodium was used for internal standardization.

For final dilution of iodide standards/internal Rh standard and probes a diluent containing 0.1% (w/v) EDTA and 0.1% (v/v) ammonia (25%) was prepared; stability 1 week at room temperature. (EDTA-ammonia imparted a stability to the diluted solutions by maintaining an alkaline pH to prevent precipitation).

Prior to measurement, all iodide standards and patients’ samples (1 ml each) were diluted 1 + 9 with diluent. Then 100 ng of internal Rh standard in an EDTA/ammonia matrix was added to each measuring solution.

All measurements were performed as 4-fold determinations (predetermined limit of acceptance was a CV<10%).

#### Quality control material

ClinChek® Urine Control, lyophil., for Trace Elements, Level I (iodine 120 μg/l), 8847 and ClinChek® Urine Control, lyophil., for Trace Elements, Level II (iodine 497 μg/l), 8848, Recipe, Munich, Germany.

### Procedure

Iodine concentrations were assayed using a Thermo-Fisher Scientific X-Series II ICP-MS in standard mode. In contrast to the Sandell-Kolthoff-method prior digestion or oxidation of the samples is not necessary. Introduced into the high temperature inductively coupled plasma source (ICP) the sample aerosol is completely desolvated and the elements converted into gaseous atoms and then ionized. Prepared samples were introduced from an autosampler (ESI SC-2) with 4 × 60-place sample racks through a concentric Mira Mist nebuliser (Burgener Research Inc., Mississauga, Ontario, Canada) and Peltier-cooled spray chamber (3 °C). Sample processing was undertaken using Plasmalab software (version 2.6.2.337; Thermo-Fisher Scientific). Quantification of iodine was carried out on the mass of the main isotope (I-127u) of the element iodine.

### Device parameters of the ICP-MS system (X-Series 2–Thermo-Fisher Scientific)


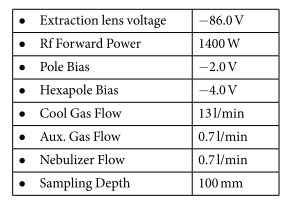


### Technical operating parameters of the method validation

Reference material for the calculation of the relative standard deviations (RSD): Seronorm™ Trace Elements Serum L-1 (SNS), 201405, and Seronorm™ Trace Elements Urine L-1 (SNU), 210605; SERO AS, Billingstad, Norway.

### Accuracy

Percentage deviation from adjusted nominal value of the certified reference material SNS (61 μg/l) and SNU (304 μg/l): 8.9% (data set = 4 × 12), from reference material ClinChek® Serum Control, lyophil., for Trace Elements, Level I (CC1P1), 8880, and Serum Control, lyophil., for Trace Elements, Level II (CC2PI), 8881, Recipe, Munich, Germany: CC1PI (45.8 μg/l) 3.2% (data set = 4 × 13), recovery 103% and CC2PI (98.4 μg/l) 1.1% (data set = 4 × 13), recovery 99%.

### Precision

Repeatability: SNS = 61 μg/l, data set = 2 × 13, RSD 2.2% and SNU = 304 μg/l, data set = 2 × 13, Relative standard deviation (RSD) 0.8%.

Intermediate precision (inter-assay CV): RSD was calculated from reference material SNS = 61 μg/l, 12 shifts, RSD 3.9% and SNU = 304 μg/l, 15 shifts, RSD 4.5%.

### Quantitation Limit

(Limit of Quantification (LOQ); empirical method):

Samples with 5; 10 and 25 μg/l iodine were measured tenfold and SD and VK calculated. LOQ was 10 μg/l, because at this concentration the CV (3.97%) is below the predetermined acceptance criteria of 10%.

### Detection Limit

(Limit of detection, LOD) was derived from LOQ ( = 1/3x LOQ = 1/3 × 10 μg/L = 3.3 μg/l).

### Range and Linearity

Within a range of 0–4000 μg/l the measured iodine results are directly proportional to the concentration of the analyte in the test sample (n = 13, r = 0.9999).

### Statistical analysis

Patient characteristics are summarized as mean and standard deviation (SD). Methods are presented with 95% confidence intervals (CI). Patients’ values obtained by the Sandell-Kolthoff-method and ICP-MS were normally distributed after logarithmic transformation (D’Agostino-Pearson test). The level of statistical significance was set at P < 0.05. All analyses were performed with the JMP® 9.0 statistical software package (SAS Institute, Cary, NC, U.S.A.) and MedCalc Statistical Software version 15.10 (MedCalc Software bvba, Ostend, Belgium). Passing-Bablok regression (Fig. 2) was used to evaluate constant and/or proportional differences between the two analytical methods, as previously described^43^,^44^. Constant differences were evaluated by calculating the intercept of the regression within the 95% CI. The Bland-Altman method (Fig. 3) plots the difference between the two methods against the geometric mean of paired results. The plot represents the mean difference in quantitative measurement of urinary iodine between the methods. The dashed lines are mean ± 1.96 SD^45^,^46^.

## Additional Information

**How to cite this article**: Haap, M. *et al*. Urinary iodine: comparison of a simple method for its determination in microplates with measurement by inductively-coupled plasma mass spectrometry. *Sci. Rep.* **7**, 39835; doi: 10.1038/srep39835 (2017).

**Publisher's note:** Springer Nature remains neutral with regard to jurisdictional claims in published maps and institutional affiliations.

## Supplementary Material

Supplementary Figure S1

## References

[b1] Teng W. . Effect of iodine intake on thyroid diseases in China. N. Engl. J. Med. 354, 2783–2793 (2006). 16807415 10.1056/NEJMoa054022

[b2] Institute of Medicine Panel on, M. Dietary Reference Intakes for Vitamin A, Vitamin K, Arsenic, Boron, Chromium, Copper, Iodine, Iron, Manganese, Molybdenum, Nickel, Silicon, Vanadium, and Zinc. National Academies Press (US) 283–314 (2001). 25057538

[b3] Koutras D. A., Papapetrou P. D., Yataganas X. & Malamos B. Dietary sources of iodine in areas with and without iodine-deficiency goiter. Am. J. Clin. Nutr. 23, 870–874 (1970). 5468832 10.1093/ajcn/23.7.870

[b4] Malamos B. . Endemic goiter in Greece: an iodine balance study in the field. J. Clin. Endocrinol. Metab. 27, 1372–1380 (1967). 4167506 10.1210/jcem-27-10-1372

[b5] Malamos B. . Endemic goiter in Greece: some new epidemiologic studies. J. Clin. Endocrinol. Metab. 32, 130–139 (1971). 4321500 10.1210/jcem-32-2-130

[b6] Pearce E. N., Andersson M. & Zimmermann M. B. Global iodine nutrition: Where do we stand in 2013? Thyroid 23, 523–528 (2013). 23472655 10.1089/thy.2013.0128

[b7] Delange F., Burgi H., Chen Z. P. & Dunn J. T. World status of monitoring iodine deficiency disorders control programs. Thyroid 12, 915–924 (2002). 12494927 10.1089/105072502761016557

[b8] Brussaard J. H., Brants H. A., Hulshof K. F., Kistemaker C. & Lowik M. R. Iodine intake and urinary excretion among adults in the Netherlands. Eur. J. Clin. Nutr. 51 Suppl 3, S59–62 (1997). 9598770

[b9] Als C. . Urinary iodine concentration follows a circadian rhythm: a study with 3023 spot urine samples in adults and children. in J. Clin. Endocrinol. Metab., Vol. 85, 1367–1369 (2000). 10770167 10.1210/jcem.85.4.6496

[b10] Wahl R., Pilz-Mittenburg K. W., Heer W. & Kallee E. [Iodine content in diet and excretion of iodine in urine]. Z. Ernahrungswiss. 34, 269–276 (1995). 8585242 10.1007/BF01625338

[b11] World Health, O. Assessment of iodine deficiency disorders and monitoring their elimination. Vol. 3rd Edition 1–98 (WHO Press, 2007).

[b12] May S. L. . Validation of a simple, manual urinary iodine method for estimating the prevalence of iodine-deficiency disorders, and interlaboratory comparison with other methods. Am. J. Clin. Nutr. 65, 1441–1445 (1997). 9129474 10.1093/ajcn/65.5.1441

[b13] Vejbjerg P. . Estimation of iodine intake from various urinary iodine measurements in population studies. Thyroid 19, 1281–1286 (2009). 19888863 10.1089/thy.2009.0094

[b14] Jooste P. L. & Strydom E. Methods for determination of iodine in urine and salt. Best Pract. Res. Clin. Endocrinol. Metab. 24, 77–88 (2010). 20172472 10.1016/j.beem.2009.08.006

[b15] Keller H. E., Doenecke D., Weidler K. & Leppla W. Kinetic studies on optimal conditions for the automated determination of low iodine concentrations by the Sandell-Kolthoff reactions. Ann. N. Y. Acad. Sci. 220, 3–14 (1973). 4512368 10.1111/j.1749-6632.1973.tb40246.x

[b16] Markou K. B. . Identification of iodine deficiency in the field by the rapid urinary iodide test: comparison with the classic Sandell-Kolthoff reaction method. Thyroid 12, 407–410 (2002). 12097202 10.1089/105072502760043495

[b17] Ohashi T., Yamaki M., Pandav C. S., Karmarkar M. G. & Irie M. Simple microplate method for determination of urinary iodine. Clin. Chem. 46, 529–536 (2000). 10759477

[b18] Grimm G. . A simple micro-photometric method for urinary iodine determination. Clin. Chem. Lab. Med. 49, 1749–1751 (2011). 21702698 10.1515/CCLM.2011.653

[b19] Hedayati M. . Rapid microwave digestion and microplate reading format method for urinary iodine determination. Clin. Chem. Lab. Med. 49, 281–284 (2011). 21143015 10.1515/CCLM.2011.053

[b20] Tang K. T. . A Simple Microplate Method with Improved Low Iodine Concentration Sensitivity in Urinary Iodine Measurement. Thyroid 25, 1173–1174 (2015). 26208060 10.1089/thy.2015.0184

[b21] Yaping Z., Dongxing Y., Jixiang C., Tianshiu L. & Huiqin C. Spectrophotometric determination of urinary iodine by flow-injection analysis with on-line catalytic digestion. Clin. Chem. 42, 2021–2027 (1996). 8969643

[b22] Gnat D. . Fast colorimetric method for measuring urinary iodine. Clin. Chem. 49, 186–188 (2003). 12507981 10.1373/49.1.186

[b23] Haglock-Adler C. J., Hurley A. & Strathmann F. G. Use of synthetic urine as a matrix substitute for standard and quality control materials in the clinical assessment of iodine by inductively coupled plasma mass spectrometry. Clin. Biochem. 47, 80–82 (2014). 25038425 10.1016/j.clinbiochem.2014.07.008

[b24] Haldimann M., Zimmerli B., Als C. & Gerber H. Direct determination of urinary iodine by inductively coupled plasma mass spectrometry using isotope dilution with iodine-129. Clin. Chem. 44, 817–824 (1998). 9554494

[b25] Caldwell K. L., Makhmudov A., Jones R. L. & Hollowell J. G. EQUIP: a worldwide program to ensure the quality of urinary iodine procedures. Accred Qual Assur 10, 356–361 (2005).

[b26] World Health, O. Urinary iodine concentrations for determining iodine status in populations. Vol. 13.1 1-5 (Vitamin and Mineral Nutrition Information System (VMNIS) 2013).

[b27] Zimmermann M. B., Jooste P. L. & Pandav C. S. Iodine-deficiency disorders. Lancet 372, 1251–1262 (2008). 18676011 10.1016/S0140-6736(08)61005-3

[b28] Delange F. Iodine deficiency as a cause of brain damage. Postgrad. Med. J. 77, 217–220 (2001). 11264481 10.1136/pmj.77.906.217 PMC1741987

[b29] Volzke H. . Ensuring Effective Prevention of Iodine Deficiency Disorders. Thyroid 26, 189–196 (2016). 26700864 10.1089/thy.2015.0543

[b30] Burgi H. Iodine excess. Best Pract. Res. Clin. Endocrinol. Metab. 24, 107–115 (2010). 20172475 10.1016/j.beem.2009.08.010

[b31] Teng X. . More than adequate iodine intake may increase subclinical hypothyroidism and autoimmune thyroiditis: a cross-sectional study based on two Chinese communities with different iodine intake levels. Eur. J. Endocrinol. 164, 943–950 (2011). 21444648 10.1530/EJE-10-1041

[b32] Luster M. . Guidelines for radioiodine therapy of differentiated thyroid cancer. Eur. J. Nucl. Med. Mol. Imaging 35, 1941–1959 (2008). 18670773 10.1007/s00259-008-0883-1

[b33] May W., Wu D., Eastman C., Bourdoux P. & Maberly G. Evaluation of automated urinary iodine methods: problems of interfering substances identified. Clin. Chem. 36, 865–869 (1990). 2357823

[b34] Dunn J. T., Crutchfield H. E., Gutekunst R. & Dunn A. D. Two simple methods for measuring iodine in urine. Thyroid 3, 119–123 (1993). 8369650 10.1089/thy.1993.3.119

[b35] Andersson M., Takkouche B., Egli I., Allen H. E. & de Benoist B. Current global iodine status and progress over the last decade towards the elimination of iodine deficiency. Bull. World Health Organ. 83, 518–525 (2005). 16175826 PMC2626287

[b36] Andersson M., Karumbunathan V. & Zimmermann M. B. Global iodine status in 2011 and trends over the past decade. J. Nutr. 142, 744–750 (2012). 22378324 10.3945/jn.111.149393

[b37] Sandell E. B. & Kolthoff I. M. Micro determination of iodine by a catalytic method. Microchimica Acta 1 9–25 (1937).

[b38] Pino S., Fang S. L. & Braverman L. E. Ammonium persulfate: a new and safe method for measuring urinary iodine by ammonium persulfate oxidation. Exp. Clin. Endocrinol. Diabetes 106 Suppl 3, S22–27 (1998). 9865549 10.1055/s-0029-1212041

[b39] Macours P. . Determination of urinary iodine by inductively coupled plasma mass spectrometry. J. Trace Elem. Med. Biol. 22, 162–165 (2008). 18565428 10.1016/j.jtemb.2008.02.003

[b40] Ford H. C. & Johnson L. A. Ascorbic acid interferes with an automated urinary iodide determination based on the ceric-arsenious acid reaction. Clin. Chem. 37, 759 (1991). 2032332

[b41] Benotti J., Benotti N., Pino S. & Gardyna H. Determination of total iodine in urine, stool, diets, and tissue. Clin. Chem. 11, 932–936 (1965). 5837839

[b42] Pearce E. N. . Urine test strips as a source of iodine contamination. Thyroid 19, 919 (2009). 19534621 10.1089/thy.2009.0120

[b43] Passing H. & Bablok. A new biometrical procedure for testing the equality of measurements from two different analytical methods. Application of linear regression procedures for method comparison studies in clinical chemistry, Part I. J. Clin. Chem. Clin. Biochem. 21, 709–720 (1983). 6655447 10.1515/cclm.1983.21.11.709

[b44] Passing H. & Bablok W. Comparison of several regression procedures for method comparison studies and determination of sample sizes. Application of linear regression procedures for method comparison studies in Clinical Chemistry, Part II. J. Clin. Chem. Clin. Biochem. 22, 431–445 (1984). 6481307 10.1515/cclm.1984.22.6.431

[b45] Bland J. M. & Altman D. G. Agreed statistics: measurement method comparison. Anesthesiology 116, 182–185 (2012). 22129533 10.1097/ALN.0b013e31823d7784

[b46] Bland J. M. & Altman D. G. Statistical methods for assessing agreement between two methods of clinical measurement. Lancet 1, 307–310 (1986). 2868172

