# Supplementary material for: Urinary iodine: comparison of a simple method for its determination in microplates with measurement by inductively-coupled plasma mass spectrometry
Source: Sci Rep. 2017 Jan 3;7:39835. doi: 10.1038/srep39835 (PMC5206638; doi:10.1038/srep39835)
Supplement: Supplementary Figure S1 [file srep39835-s1.pdf]

Urinary iodine: comparison of a simple method for its determination in microplates with measurement by inductively-coupled plasma mass spectrometry

Michael Haap[1], Heinz Jürgen Roth[2], Thomas Huber[2], Helmut Dittmann[3] and Richard Wahl[1]

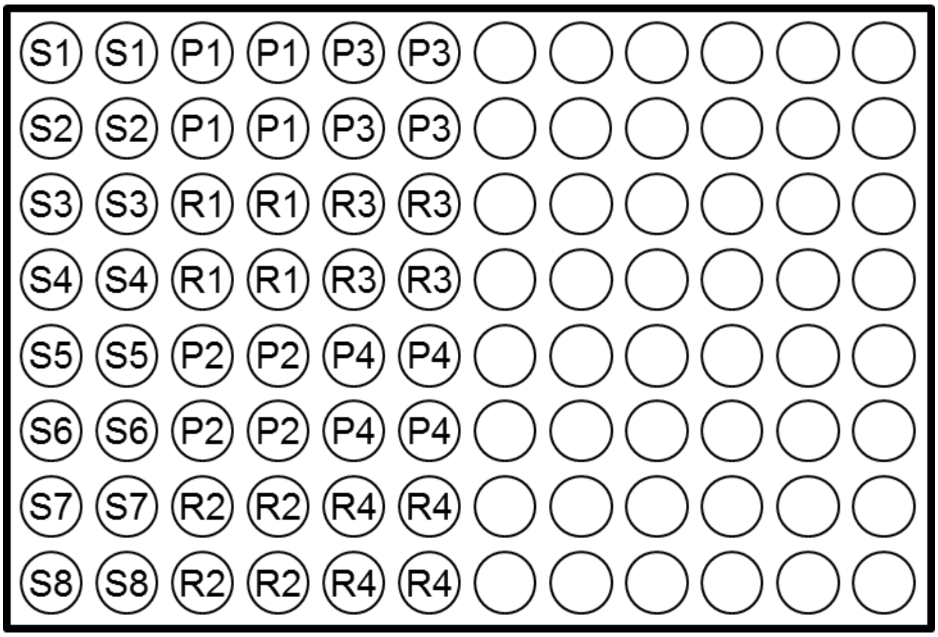

Supplementary Figure S 1: Arrangement of samples in the microplate. S1-S8 standards, P patients, R Recovery of iodine in the patient`s samples. The remaining vials can be filled with additional patient samples.
